# Willingness of college students aged 10 to 24 years to get vaccinated against COVID-19 disease: a cross-sectional study in South-East Nigeria

**DOI:** 10.4314/ahs.v23i2.4

**Published:** 2023-06

**Authors:** Awoere T Chinawa, Edmund N Ossai, Chinedu Ugwunna Nwachukwu, Ndubuisi A Uwaezuoke, Ann E Aronu, Josephat M Chinawa

**Affiliations:** 1 Department of Community Medicine, Enugu State University College of Medicine; 2 Department of Paediatrics, College of Medicine, University of Nigeria Enugu Campus, Nigeria; 3 Department of Community Medicine, College of Health Sciences, Ebonyi State University; 4 Department of Community Medicine, University of Nigeria Enugu Campus, Nigeria

**Keywords:** Willingness, vaccination, college adolescents, COVID-19, knowledge, Enugu

## Abstract

**Background:**

Willingness to be vaccinated against COVID-19 is a topical issue that may change the course and distribution of the pandemic in the country.

**Objectives:**

This study was aimed to determine the willingness to receive the COVID-19 vaccine among college adolescents and associated factors.

**Methodology:**

This was a cross-sectional study carried out among one thousand college adolescents in six secondary schools in Enugu from April to August 2021. A pretested, self-administered questionnaire was used for data collection. Data entry and analysis were done using IBM Statistical Package for Social Sciences (SPSS) statistical software version 25. Descriptive statistics were used to describe college adolescents' characteristics. Categorical variables were reported as frequencies and percentages. Predictors of willingness to vaccinate were assessed using binary logistic regression.

**Results:**

A minor proportion of the respondents, 13.2% (153) were willing to receive the COVID-19 vaccine. The respondents who were males were 1.6 times more willing to receive the COVID-19 vaccination when compared with those who were females. (AOR=1.6, 95%CI: 1.1- 2.3). The respondents who were aware they could be infected with COVID-19 were twice more likely to receive COVID-19 vaccination when compared with those who felt they could not be infected. (AOR=2.0, 95%CI: 1.1-3.1). The respondents who had good knowledge of COVID-19 vaccination were 2.2 times more likely to receive COVID-19 vaccination when compared with those who had poor knowledge. (AOR=2.2, 955CI: 1.5-3.3)

**Conclusion:**

A small fraction of college adolescents were willing to receive the COVID-19 vaccine. Male gender, knowledge of vaccine and possibility of transmitting infection are predictors of willingness to receive the vaccination.

## Introduction

Corona virus 2019 (COVID-19) pandemic caused by severe acute respiratory syndrome coronavirus 2 (SARS-CoV-2) was first seen in Wuhan, China in December 2019 1. Since then, there has been a rapid spread of the virus around the world. As of 7^th^ January 2022, Covid-19 has spread around the world, with nearly 300 million cases and more than five million deaths across almost 200 countries 2. In Africa, more than 10 million cases with over 230,000 deaths have been recorded. In Nigeria, till date, over 24 million cases have been confirmed with more than 3 thousand deaths 3.

In the absence of appropriate vaccines and established pharmacological intervention, the world has made efforts to contain the virus through non-pharmacological methods. These include the enforcement of quarantine and lock downs, social distancing, use of face masks, hand washing and travel restrictions. There is a well-coordinated effort in the developed countries especially in Europe and the US in developing a COVID-19 vaccine that is safe, affordable and effective, with an estimate of more than 100 candidate vaccines currently in different development stages 4 and numerous candidate vaccines already in clinical trials 5,6. Currently, more than 50% of people have been fully vaccinated on every continent apart from Africa, where less than 10% have been vaccinated. However, in Nigeria, the National Primary Healthcare Development Agency has approved the COVID-19 vaccination of adolescents 7,8.

Though the western world has secured more vaccine doses, the low-income countries are relying on a global plan known as COVAX that seeks to make vaccines accessible to everyone in the world 2.

It is important to note that in protecting society as a whole and decrease household transmission of COVID-19, it is expedient to vaccinate children and adolescents, as well as adults 2. The American Academy of Pediatrics (AAP) has recommended COVID-19 vaccination for all children and adolescents who were 12 years of age and above, Given the need for rapid uptake of COVID-19 vaccines, the AAP co-administered the routine childhood and adolescent immunizations with COVID-19 vaccines especially for children and adolescents 9-11. Zimet et al 12, noted the need to include children and adolescents in COVID-19 vaccine clinical trials and the necessity of including minors as important targets for vaccination. This is crucial because it will also protect older adults with whom they interact through herd immunity. Furthermore, the availability of COVID-19 vaccines will allow children, adolescents, and adults to lead a normal life that was enjoyed in the pre- COVID era.

The willingness of the adolescent child to accept vaccination against COVID-19 is a difficult task 13. For instance, a recent poll in the US showed that only 50% of Americans plan to get the vaccine 13, while another study revealed that two-thirds of Americans say they will not get the COVID-19 vaccine when it is first available, and 25% say they will never get it. 14 This hesitancy may be due to the significant amount of misinformation about the COVID-19 vaccine 14.

A search across literature yielded very little information on the willingness of the college adolescents to accept COVID-19 vaccination 15. Most studies are mainly among the adult population. This study was aimed to determine the willingness of the adolescent college student to accept the COVID vaccine and its associated factors. This is the first time this study has been carried out in this locale. The study can however inform policies to inform the roll-out of vaccination in that age group.

## Methods

### Study Area

The study was carried out in six secondary schools located in Enugu city. The schools were drawn from a frame of private and public schools.

### Study Design

This was a cross-sectional study among college adolescents attending secondary schools in Enugu city. This study was conducted from April to August 2021.

### Study population

A total of one thousand, one hundred and sixty-two college adolescents from six secondary schools were enrolled in the study. Every detail of the study was explained to the adolescent students and information in the questionnaire was described to them in a simple language.

### Inclusion Criteria

Adolescents who gave consent/assent and who were seen in the class at the time of the study.

### Exclusion Criteria

Adolescents without consent/assent were excluded from the study

### Sampling technique

A two-stage sampling technique was used to select the students for inclusion in the study. There are 468 secondary schools in Enugu metropolis including public and private secondary schools. Enugu metropolis is made up of three local government areas including Enugu East, Enugu South and Enugu North local government areas. The list of schools in the three local government areas in the metropolis was made and ranked in terms of the number of students in the schools. This was done separately for public and private secondary schools in each of the three local government areas. The first five schools based on this ranking in the three local government areas were selected. Using a simple random sampling technique of balloting one public and one private secondary school was selected in each of the three local government areas in the metropolis. This served as the first stage.

In the second stage, a list of all students in the senior secondary classes one, two and three and junior secondary class three was made. On each day of data collection, the number of students in the four classes in each of the six selected schools who were present served as the sampling frame. The sampling interval was obtained by dividing this number by the sample size of 200, which was the estimated number of students to be contributed to the study by each of the selected schools. So, every nth student was recruited for the study, based on the sitting arrangement of the students in the four classes on each day of data collection. On each occasion, the index student was selected using a simple random sampling technique of balloting

### Ethical Consideration and consent

Ethical approval for the study was obtained from the Health Research and Ethics Committee of the University of Nigeria Teaching Hospital Ituku-Ozalla Enugu. The students who were 18 years and above were required to sign a written informed consent form before participating in the study. Assent was obtained from the students who were less than 18 years old and written informed consent was obtained from their parents. This was done by giving the consent form to the students for their parents to sign after which the consent form was returned the following day by the students. Participation in the study was voluntary and participants were assured that there would be no victimization of anyone who did not want to participate or who decided to withdraw after giving consent.

### Study tool

Information was obtained from the students using a pre-tested questionnaire which was designed by the researchers. The questionnaire was interviewer-administered to students especially those in junior secondary three and senior secondary one student. The questionnaire was self-administered to the students who were in senior secondary three class.

### Sample Size Determination

The minimum sample size for the study was determined using the sample size formula for single proportions 17. A sample size of 1162 respondents were included in the study based on a type 1 error (a) of 0.05, a tolerable margin of error of 0.05 and the proportion of 50.1% of children and adolescents who were willing to receive COVID-19 vaccination in a study in a large school survey in England 18.

### Study Instrument

A pretested and validated self- administered questionnaire was used in the study. Information obtained included: demography, willingness to accept vaccination and predictors of willingness to be vaccinated.

### Definition of terms

Adolescence: This is the interval between childhood and adulthood. Based on current studies on adolescent growth and function, the age range of 10–24 years was used in this study 19,20.

**Willingness to be vaccinated:** This is defined as the readiness or preparedness to receive a vaccine after considering the benefits, severity, barriers and efficacy of receiving such vaccine 21–23.

### Data analysis

Data entry and analysis were done using IBM Statistical Package for Social Sciences (SPSS) statistical software version 25. Categorical variables were summarized using frequencies and proportions while continuous variables were summarized using mean and standard deviation. Chi-square test and multivariate analysis using binary logistic regression were used in the analysis and the level of statistical significance was determined by a p-value of <0.05.

The outcome measure of the study was the willingness to receive COVID vaccination among the respondents. The response to this variable was a five-point Likert scale of strongly disagree, disagree not sure, agree and strongly agree. The responses were categorized into two, agree and strongly agree denoted as Yes, disagree, strongly disagree and not sure classified as NO.

Knowledge of COVID-19 vaccination was determined using nine variables. For each respondent, a correct answer to each of the variables attracted a score of one while an incorrect answer was scored zero. Respondents who scored ≥50% of correct answers to the nine variables were regarded as having good knowledge of COVID-19 vaccination while those that scored <50% were classified as having poor knowledge.

Multivariate analysis using binary logistic regression was used to determine factors predicting the willingness to receive COVID-19 vaccination. Variables that had a p-value of <0.2 in bivariate analysis, were entered into the logistic regression model to determine the predictors of the willingness of the respondents to receive COVID-19 vaccination. Results of logistic regression analysis were reported using an adjusted odds ratio and 95% confidence interval and the level of statistical significance was determined by a p-value of <0.05.

In determining factors affecting willingness to receive COVID-19 vaccination, the age of the respondents was categorized into two, those less than 15 years and those 15 years and above.

The family socio-economic status index was developed using Principal Component Analysis, (PCA) in STATA statistical software version 12. The input to the PCA included family ownership of eleven household items that included a gas cooker, television, electric iron, refrigerator, cable television, electric fan, air conditioner, motor vehicle, generator, microwave oven and washing machine. For the calculation of distribution cut points, quartiles were used. Each respondent was assigned the wealth index score of his/her family. The quartiles were Q1 = Poorest, Q2= The Very Poor, Q3= The Poor, and Q4= The Least Poor. The quartiles were further dichotomized into low socio-economic class comprising the poorest and very poor and high socio-economic class made up of the poor and least poor groups.

## Results

Table 1 shows the socio-demographic characteristics of the respondents. The mean age of the respondents was 15.0±1.8 years. The majority of the respondents, 56.8%, (660) were in the age group of 15-19 years while the least proportion, 1.5% (18), were 20 years and above. A higher proportion of the respondents, 59.4% (690), were males. The highest proportion of the respondents, 28.9% (336), were in senior secondary three class while the least proportion, 21.9% (254), were in senior secondary one class.

**Table 1:** Socio-demographic characteristics of respondents

Variable	Frequency (n=1162)	Percent (%)
**Age of respondents**		
Mean±(SD)	15.0±1.8	

**Age of respondents in groups**		
<15 years	484	41.7
15-19 years	660	56.8
≥20 years	18	1.5

**Gender**		
Male	690	59.4
Female	472	40.6

**Marital status**		
Single	1119	96.3
Married	43	3.7

**Class of study**		
Junior secondary class 3	308	26.5
Senior secondary class 1	254	21.9
Senior secondary class 2	264	22.7
Senior secondary class 3	336	28.9

**Educational attainment of Father**		
No formal education	52	4.5
Primary education	60	5.2
Secondary education	215	18.5
Tertiary education	835	71.9

**Educational attainment of Mother**		
No formal education	37	3.2
Primary education	42	3.6
Secondary education	182	15.7
Tertiary education	901	77.5

**Employment status of Father**		
Unemployed	57	4.9
Self-employed	575	49.5
Salaried employment	530	45.6

**Employment status of Mother**		
Unemployed	85	7.3
Self-employed	449	38.6
Salaried employment	628	54.0

**Family socio-economic status**		
Low socio-economic class	583	50.2
High socio-economic class	579	49.2

Table 2 shows the awareness of the COVID-19 vaccine among the respondents. The Majority of the respondents, 81.2% (944), were aware of the availability of COVID-19 vaccine. The major sources of information on the COVID-19 vaccine among the respondents included television, 85.9% (811); internet/social media. 83.4% (787) and parents/family, 80% (755). A minor proportion, 14.5% (169), were aware they could be infected with COVID-19. A minor proportion, 19.7% (229), were aware of someone infected with COVID-19. More than a quarter of the respondents, 26.2% (305), were aware of someone who died from COVID-19.

**Table 2:** Awareness of COVID-19 vaccine

Variable	Frequency (n=1162)	Percent (%)
**Aware of COVID-19 vaccine**		
Yes	944	81.2
No	218	18.8

Source of information on COVID-19 vaccine**		
Television	811	85.9
Internet/social media	787	83.4
Parents/family	755	80.0
Friends	662	70.1
Radio	613	64.9
Religious gathering	480	50.8
Health workers	437	46.3
Newspaper	432	45.8
Posters and banners	417	44.2

Community Meeting	202	21.4

**I am a high priority group for COVID-19 vaccine in Nigeria**		
Yes	92	7.9
No	1070	92.1

**I could be infected with COVID-19**		
Yes	169	14.5
No	993	85,5

**I have been tested for COVID-19**		
Yes	100	8.6
No	1062	91.4

**Aware of anyone that have tested for COVID-19**		
Yes	284	24.4
No	878	75.6

**Aware of anyone infected with COVID-19**		
Yes	229	19.7
No	933	80.3

**Aware of anyone that died from COVID-19**		
Yes	305	26.2
No	857	73.8

** multiple responses encouraged

Table 3 shows the willingness to receive the COVID-19 vaccine among the respondents. A minor proportion of the respondents, 13.2% (153), were willing to receive the COVID-19 vaccine. The major reasons for not being willing to receive the COVID-19 vaccine included not being sure of the content of the vaccine, 33.2% (240), not being sure of the source of the vaccine, 28.7% (207), and fear of complications,18.7% (135).

**Table 3:** Willingness to receive COVID-19 vaccination

Variable	Frequency (n=1162)	Percent (%)
**Willing to receive COVID-19 vaccination**		
Yes	153	13.2
No	1009	86.8

Reason for not being willing to receive COVID-19 vaccination**	**(n=722)**	
Unsure of the content of the vaccine	240	33.2
Unsure of source of vaccine	207	28.7
Fear of complications	135	18.7
Religious reasons	50	6.9
Cultural reasons	20	2.8
No specific reason	70	9.7

** Strongly disagree, disagree

Table 4 shows the knowledge of COVID-19 vaccination among the respondents. Majority of the respondents 70.0% (913), knew that vaccination is a good measure to prevent diseases. Majority, 62.0% (720), were aware that one may need to wear a face mask even after receiving COVID-19 vaccine. A minor proportion, 37.0% (430), knew that vaccines have been produced for the prevention of COVID-19. Less than half of the respondents, 49.3% (573), had good knowledge of COVID-19 vaccination.

**Table 4:** Knowledge of COVID-19 vaccination

Variable	Frequency (n=1162)	Percent (%)
**Vaccination is a good measure to prevent diseases**		
Yes (Correct)	913	70.0
No	349	30.0

**COVID-19 vaccine will be of immense help in the fight against the disease**		
Yes (Correct)	567	48.8
No	595	51 .2

**One is expected to wear a face mask even after receiving the COVID-19 vaccine**		
Yes (Correct)	720	62.0
No	442	38.0

**All vaccinations are beneficial to man except COVID-19 vaccine**		
No (Correct)	475	40.9
Yes	687	59.1

**There is no disease called COVID-19 hence COVID-19 vaccine is false**		
No (Correct)	732	63.0
Yes	430	37.0

**There is no COVID-19 in Nigeria hence no need for COVID-19 vaccine in Nigeria**		
No (Correct)	600	51.6
Yes	562	48 .4

**Side effects of COVID-19 vaccine have been reported after administering the vaccine**		
Yes (Correct)	365	31.4
No	797	68 .6

**Vaccines have been developed for the prevention of COVID-19**		
Yes (Correct)	430	37.0
Wrong	732	63.0

**Nigeria is interested in providing COVID-19 vaccine to its citizens**		
Yes (Correct)	365	31.4
Wrong	797	68 .6

**Knowledge of COVID-19 vaccination**		
Good	573	49.3
Poor	589	50.7

Table 5 shows factors associated with willingness to receive COVID-19 vaccination. The respondents who were males were 1.6 times more willing to receive COVID-19 vaccination when compared with those who were females. (AOR=1.6, 95%CI: 1.1- 2.3). The respondents who were aware they could be infected with COVID-19 were twice more likely to receive COVID-19 vaccination when compared with those who felt they could not be infected. (AOR=2.0, 95%CI: 1.1-3.1). The respondents who were aware of someone infected with COVID-19 were about twice more likely to receive COVID-19 vaccination when compared with those who knew no one infected with COVID-19. (AOR=1.9, 95%CI:1.1-3.2). The respondents who had good knowledge of COVID-19 vaccination were 2.2 times more likely to receive COVID-19 vaccination when compared with those who had poor knowledge. (AOR=2.2, 955CI: 1.5-3.3)

**Table 5:** Factors affecting willingness to receive COVID-19 vaccine

Variable	Willing to receive COVID-19 vaccine (n=1162)		p value on bivariate analysis	AOR (95%C1)**
	Yes N (%)	No N (%)		
**Age of respondents**				
<16 years	98 (13.9)	607 (86.1)	0.358	NA
≥16 years	55 **(12.0)**	402 (88.0)		

**Gender**				
Male	103 (14.9)	587 (85.1)	0.032	1.6 (1.1- 2.3)
Female	50 (10.6)	422 (89.4)		

**Marital status**				
Single	145 (13.0)	974 (87.0)	0.283	NA
Married	8 (18.6)	35 (81.4)		

**Class of study**				
Junior secondary class 3	38 (12.3)	270 (87.7)	0.051	1.1 (0.6- 1.7)
Senior secondary class 1	30 (11.8)	224 (88.2)		0.9 (0.5- 1.6)
Senior secondary class 2	48 (18.2)	216 (81.8)		1.7 (1.1- 2.7)
Senior secondary class 3	37 (11.0)	299 (89.0)		1

**Educational attainment of Father**				
Tertiary education	119 (14.3)	716 (85.7)	0.081	1.1 (0.6- 1.8)
Secondary education and below	34 (10.4)	293 (89.6)		1

**Educational attainment of Mother**				
Tertiary education	125 (13.9)	776 (861)	0.186	1.2 (0.7- 2.3)
Secondary education and below	28 (10.7)	233 (89.3)		1

**Employment status of Father**				
Unemployed	6 (10.5)	51 (89.5)	0.342	NA
Self-employed Salaried employment	69 (12.0)	506 (88.0)		

**Employment status of Mother**				
Unemployed	10 (11.8)	75 (88.2)	0.267	NA
Self-employed	51 (11.4)	398 (88.6)		
Salaried employment	92 (14.6)	536 (85.4)		

**Family socio-economic status**				
Low socio-economic class	74 (12.7)	509 (87.3)	0.632	NA
High socio-economic class	79 (13.6)	500 (86.4)		

**One could be infected with COVID-19**				
Yes	42 (24.9)	127 (75.1)	<0.001	2.0 (1.3- 3.1)
No	111 (11.2)	882 (88.8)		1

**Been tested for COVID-19**				
Yes	20 (20.0)	80 (80.0)	0.035	1.5 (0.9- 2.7)
0No	133 (12.5)	929 (87.5)		1

**Aware of someone that have tested for COVID-19**				
Yes	46 (16.2)	238 (83.8)	0.082	0.7 (0.4- 1.1)
No	107 (12.2)	771 (87.8)		1

**Aware of someone infected with COVID-19**				
Yes	51 (22.3)	178 (77.7)	<0.001	1.9 (1.1- 3.2)
No	102 (10.9)	831 (89.1)		1

**Aware of someone that died from COVID-19**				
Yes	63 (20.7)	242 (79.3)	<0.001	1.5 (0.9- 2.3)
No	90 (10.5)	767 (89.5)		1

**Knowledge of COVID-19 vaccination**				
Good	105 (18.3)	468 (81.7)	<0.001	2.2(1.5- 3.3)
Poor	48 (8.1)	541 (91.9)		1

** Adjusted odds ratio, 95% confidence interval

NA Not applicable

## Discussion

This study showed that 81.2% of college adolescents were aware of the availability of the COVID-19 vaccine. The result obtained in this study was higher than that of Enitan et al 31 who noted that 39.0% of their subjects had a poor perception of the COVID-19 Vaccine availability. Their study however, was among the adult population. The high level of awareness from this study could partly be explained by the adolescents' dependence on information on the COVID-19 vaccine from television and internet/social media.

This study showed that 13.2% of the adolescents are willing to receive the COVID-19 vaccine. This study was probably the lowest seen in all literature. For instance, studies on the willingness to be vaccinated across seven European countries revealed that 74% of adults were willing to get a COVID-19 vaccine when available 32. Similarly, data from 27,000 UK students who were between nine and 18 years revealed that majority of older school-aged students are willing to be vaccinated while the rates for adolescents between 16 and 17 years were as high as adults with lower rates in younger students 15.

Besides, a study also showed that only 28% of participants would get a vaccine against COVID-19 when it becomes available 33. Furthermore,86% of respondents in another study reported a willingness to receive a vaccine for COVID-19 33-35.

Very high levels of willingness to be vaccinated against COVID-19 were also noted in France where values of 77.6% were obtained 36. A study of 13,426 people in 19 countries to determine potential acceptance rates and factors influencing acceptance of a COVID-19 vaccine also noted that 71.5% of participants reported they would likely take the COVID-19 vaccine 37.

The major reasons for not being willing to receive the COVID-19 vaccine seen in this study included not being sure of the content of the vaccine, not being sure of the source of the vaccine, and fear of complications. Anecdotal evidence has infertility as a major concern. Although adolescents may want to protect themselves against infection, they also fear possible side effects from vaccination. They have doubts about the safety of the vaccine, given the rapid pace of trial and development, and whether possible side effects have been adequately studied 38. Britain has reported numerous cases of anaphylaxis among people who have received the Pfizer vaccine 39. Bell's palsy has also been noted to be linked to the COVID-19 vaccine in Switzerland 39. Besides, according to CDC, 11 cases have been reported since the vaccine was inaugurated in the US. Notwithstanding, a three-year review of vaccine-related adverse effects in the U.S. national vaccine database found that anaphylaxis after vaccination is rare, occurring at a rate of about 1.31 per million doses of vaccine administered. They noted rather that these adverse reactions were noted in 85% of people with a history of allergies 39. Unwillingness to receive vaccination by college adolescents could further be explained by the poor knowledge of COVID-19 vaccination. This is shown in our study where college adolescents who had good knowledge of COVID-19 vaccination were 2.2 times more likely to receive the COVID-19 vaccination when compared with those who had poor knowledge. Of the college adolescents, in this study,62.0%, were aware that one may need to wear a face mask even after receiving the COVID-19 vaccine. Wearing face masks is crucial even after vaccination. Though the novel COVID-19 vaccine may prevent serious complications from COVID-19 but much is not known if vaccination will curb the spread of the coronavirus. vaccinated individuals may be silent spreaders of the virus, they have a high tendency to keep it circulating in communities, putting those without vaccination at a greater risk 40,41. The respondents who were males were 1.6 times more willing to receive COVID-19 vaccination when compared with those who were females. A study in China also noted that the quest for men to get vaccinated could be explained by the findings in a recent study that showed that women's samples had more antibodies which may have an impact on their immune response compared to the men's samples 42. Pulcini et al 43 also noted that more men accepted getting vaccinated compared to women in their study and they attributed this to the increased risk perception of disease in men compared to women 42,44. Other studies have also shown that females are more likely to be unwilling to be vaccinated against COVID-19 which is also consistent with the work in France, 45 and Australia 45

Furthermore, Lazarus et al 46 noted no gender correlates on vaccine acceptance in their study. The sample size used in this present study is quite large and this may explain the difference in gender as regards willingness to accept vaccine when compared to the studies above.

Surprisingly, age, marital status, socioeconomic class, employment status and level of education of parents did not significantly affect the willingness of the college adolescents to get vaccinated in this study. This was contrary to the study of Lazarus et al [46] who noted that people in a higher socio-economic class and higher income were most likely to accept a vaccine than those with a lower income.

Further analysis from this study revealed that the college adolescents who were aware they could be infected with COVID-19 were twice more likely to receive COVID-19 vaccination when compared to those who felt they could not be infected. Besides, college adolescents who were aware of someone infected with COVID-19 were about twice more likely to receive COVID-19 vaccination when compared to those who were not.

Misinformation and disinformation are strong tools for vaccine scepticism. This could frustrate all efforts to end the coronavirus pandemic. There is therefore an urgent need for adequate public health enlightenment on the role of the vaccine in curbing COVID-19 pandemic. This will help in prompt deployment and acceptance of the vaccine. 45 Dissemination of accurate information about preventing COVID-19 is important for vaccine compliance 45. Health workers, religious leaders and school authorities where these adolescents are domiciled play an important role in disseminating accurate information about the vaccine 45.

## Conclusion

A small fraction of college adolescents was willing to receive the COVID-19 vaccine. Male gender, good knowledge of vaccine and possibility of transmitting infection are predictors of willingness to receive the vaccination.

## Recommendation

College adolescents are not willing to receive vaccination against COVID-19. Dissemination of accurate information on the prevention of COVID-19 is important for vaccine compliance. The governmental and non-governmental organizations, religious leaders and school authorities where these adolescents are domiciled must play a role in disseminating accurate information about the vaccine.

## Limitation

The Authors did not include private and public secondary schools as independent variables when determining factors associated with willingness to receive COVIID-19 vaccination. Also, because of the classes of students included in the study, (junior secondary three and senior secondary one to three classes) individual classes were used in factors associated with willingness to receive COVID-19 vaccination.

## Declaration

### Availability of data and materials

We have attached the questionnaire and the excel sheets/ SPSS of the work.
